# 以新视角观察p53家族在肺癌发生及治疗中的独特作用

**DOI:** 10.3779/j.issn.1009-3419.2013.08.06

**Published:** 2013-08-20

**Authors:** 文彦 黄, 凯珊 刘

**Affiliations:** 510632 广州，暨南大学医学院病理系 Department of Pathology, Medical College, Ji'nan University, Guangzhou 510632, China

**Keywords:** P53/P63/P73, 肺肿瘤, 化疗敏感性, 靶向治疗, P53/p63/p73, Lung neoplasms, Chemosensitivity, Target treatment

## Abstract

P53作为转录因子，其转录激活功能维持了基因组的稳定性，对防止肿瘤的形成起着重要作用，是目前研究得最为广泛、深入的抑癌基因，被称为“基因卫士”。P53家族的成员p63、p73与p53在DNA结合结构域上有高度的同源性，某些p53家族亚型可以与p53-反应基因相结合起着转录激活的作用，另外一些则起着负性调节作用。肺癌是世界上患病率最高的恶性肿瘤之一，p53家族成员在肺癌中的异常表达与肺癌的发生有密切联系，并导致不良的预后及对放疗、化疗的抵抗。对p53家族成员在肺癌致病机制的深入研究可有助于为临床提供合理的化疗方案及靶向治疗策略。本文着重回顾总结p53家族成员在肺癌发生、化疗敏感性以及肺癌靶向治疗中的独特的作用。

## Abstract (en)

The transcription activation of p53 plays an important role in the maintenance of genetic stability. P53 is an intensive study tumor suppressor, which has been called the "gene guider". The p53 family members p63, p73 have high homologous sequence with p53. Some of them can bind to the p53-responsive genes and transcript the downstream genes. Human lung cancer is one of the most common malignant tumors in the world. Abnormality of the *p53* gene is the signifcant event in lung cancers, which leads to the poor prognosis and the resistance of chemotherapy. A deep understanding of the relationship between p53 family members and lung cancers can provide a more reasonably targeted clinical approach. Tis paper will focus on the special function of p53 family members in the development, chemosensitivity and target treatment of lung cancer.

*P53*基因开始时被认为是原癌基因，后来的研究才得以证实*p53*其实是抑癌基因^[1]^。人类*p53*基因定位于17号染色体的短臂，有11个外显子，转录成2.5 Kb mRNA，编码393个氨基酸，分子量为53 kDa。P53蛋白质结构可分为N末端的转录激活(transaction, TA)结构域，DNA结合结构域及四聚体寡聚化结构域，根据N末端不同的启动位点及C末端α、β、γ3种不同剪切可分为9种不同的亚型，其N端含TA结构的亚型有转录激活的功能，N端为delta亚型则丧失转录激活功能，起着显性失活的作用。P63、p73与p53在DNA结合结构域上有高度的同源性，允许p63、p73与p53-反应基因相结合起着转录激活的作用。目前研究发现p63有6种亚型，p73有29种亚型^[2]^。P53作为转录因子，在细胞受到损伤时可以直接激活周期依赖激酶抑制剂，p21等诱导细胞周期阻滞，或者通过激活促凋亡基因如*bax*、*puma*、*scotin*等诱导细胞凋亡^[3]^。全长的p53很快被MDM2等降解，在外来刺激强烈的情况下，*p53*会发生突变或其它变化，使p53丧失转录激活的功能致使肿瘤发生并具有增加恶性、患者的不良生存及对治疗的抵抗等特征。

肺癌源于支气管上皮，可分为小细胞肺癌(small cell lung cancer, SCLC)及非小细胞肺癌(non-small cell lung cancer, NSCLC)两大类，其中NSCLC又包括鳞癌、腺癌和大细胞癌。SCLC对首次化疗较NSCLC敏感，但更容易复发，复发后产生化疗抵抗，使预后不良^[4]^。肺癌的发病率很高，多数与吸烟相关，虽然外科手术和放化疗的治疗技术都在进步，但肺癌的预后仍然较差。肺癌的发生涉及多种基因多步骤的恶性转化过程，其中p53家族成员起着重要作用。

## 1 P53家族成员在肺癌发生中的作用

### 1.1 *P53*突变与肺癌的发生

*P53*突变发生在许多人类肿瘤中，多达50%的人类肿瘤出现*p53*的等位基因突变^[5, 6]^，与其它抑癌基因不同的是，*p53*主要发生错义突变，导致单个氨基酸的改变，从而在不同的水平上影响着*p53*的转录活性^[7]^，而在人类肺癌中超过90%的*p53*错义突变会导致其异常积累，引起突变蛋白质的稳定性增加^[5]^。*P53*突变常导致表达一个拥有"获得性功能"(gain of function, GOF)的稳定的突变蛋白质。在肺癌中，有33%的腺癌和70%的SCLC发生*p53*突变，而且，*p53*突变的肺癌细胞预后不良并对化疗药物和放疗更加抵抗，许多不同的表型归因于GOF的活化，包括增加致癌性，增加肿瘤转移和侵袭的能力，降低对化疗药物的敏感性，增加生存率，增强运动能力等^[8]^。

在肺癌的细胞系和病理标本中经常都可以检测出17号染色体*p53*基因的位点上发生杂合性缺失(loss of heterozygosity, LOH)，研究表明尽管有*p53*突变基因的积累，但在p53功能丧失的肺癌细胞中重新加入正常的p53可以恢复其肿瘤抑制能力，这表明LOH可能与肺癌的发生相关^[9]^。免疫组化的研究^[10]^结果表明，在NSCLC临床标本中鳞癌的*p53*突变频率最高于腺癌。*P53*突变常发生在外显子5-8的区域，运用单链构象多态性分析及PCR扩增片段的变性梯度凝胶电泳技术可检测*p53*的突变位点^[11, 12]^。P53突变是肺癌发生的早期事件，在支气管癌早期就能检测出分子损伤，而*k-ras*癌基因突变则在支气管癌中出现较迟，通常不能在癌前病变检测出来^[13]^。

### 1.2 P63、p73与肺癌的发生

P63有P1、P2两个启动子和α、β、γ三个不同的C末端剪切，共有TA p 6 3和ΔNp63两大类共6种蛋白质；p73中TAp73、外显子2p73 (Exons2p73，EX2p73)、外显子2/3p73(Exons2/3p73，EX2/3p73)、ΔN'p73的启动子为P1，C末端有α、β、γ、δ、ε、ζ、η7种不同的剪切，ΔNp73的启动子为P2，缺乏DNA结合与C末端的结构域，所以p73共编码29种蛋白质^[2, 3]^。*P63*、*p73*与*p53*不同的是很少发生突变，而是通过表达有N-末端删节的ΔNp63、ΔNp73起到抗凋亡的作用，与之相反的是有转录激活功能的TAp63、TAp73，后两者与p53都起着细胞周期阻滞及诱导凋亡的功能^[14, 15]^。ΔNp63、ΔNp73可通过显性失活的作用抑制其TA亚型或者p53的诱导凋亡作用，近年来发现p63、p73Δ:TA的比例，而不是单个的亚型，在肿瘤的发生中起着一定的作用。在膀胱癌、卵巢癌、乳腺癌和大肠癌中p63、p73的异常表达尤为明显^[16]^。

与其它恶性肿瘤一样，肺癌中p63、p73异常表达的作用及地位相当复杂，目前仍比较有争议。88%的鳞癌，42%的大细胞肺癌及11%的腺癌出现*p63*基因的扩增，主要改变的亚型是ΔNp63α，并且主要表达在正常支气管上皮及鳞癌中，少有表达在正常肺组织及腺癌中，提示了*p63*基因序列的扩增可能参与了肺鳞癌的发展。在一项组织芯片的检测中得出同样的结论，并且提示p63免疫组化阳性反应与3号染色体长臂27-29区域的DNA扩增呈显正相关，*p63*基因的扩增与肺鳞癌发生和发展关系密切^[17, 18]^。另外基因芯片检测也显示肺鳞癌中有3q26-29明显扩增^[19]^。一项关于肺癌患者标本中ΔNp73的检测显示，有77/132(58.3%)肺癌细胞质中检测出Δ Np73的阳性表达，且预后不良^[20]^。NSCLC患者的肺癌标本实验显示，p63、p73中N末端Δ:TA的比例发生失调，特别是ΔNp63明显的上调而TAp63却明显地下调，同样地，Δ2p73和Δ2/3p73都上调了，而ΔNp73和ΔN'p73都下调了，提示了p63、p73中Δ:TA表达的异常可能对NSCLC的发生起着一定的作用^[21, 22]^。*P73*基因的第二外显子的非编码区有多态性表现，会改变p73的翻译效率，相对于GC/GC基因型，GC/AT与AT/AT基因型对增加肺癌的风险有统计学意义^[23]^。

## 2 P53家族成员对肺癌化疗敏感性的影响

肿瘤细胞有无限增殖的潜能，并且通过各种机制逃避凋亡。DNA损伤药物表现出明显的肿瘤细胞毒性，运用DN A损伤药物治疗肿瘤可以使肿瘤细胞实现多种方式的细胞死亡，如衰老、有丝分裂失败、凋亡等。P53的状态在这些DNA损伤药物对肿瘤细胞的毒性中占有重要地位。TAp63/ΔNp63、TAp73/ΔNp73的比例及突变型*p53* (mutant type p53, MTp53)的水平对化疗的效果有一定影响。对ΔNp63、ΔNp73、MTp53实施干扰能够增强化疗的敏感性及降低肿瘤侵袭和转移的能力。P53的不同亚型在诱导凋亡中发挥着不同的生物学功能，p53β更易于结合到*p21*和*bax*，而不易结合到*MDM2*，共转染p53β可轻微增加p53介导的凋亡^[24, 25]^。

### 2.1 p53与肺癌的化疗敏感性

MTp53可导致NSCLC增加化疗抵抗。NSCLC患者的临床病理标本中MTp53的表达与以铂类药物为基础的化疗抵抗有密切的联系^[26, 27]^。重组腺病毒治疗在临床试验中证实可增强化疗的敏感性，在NSCLC实体瘤中注射野生型p53(wild type p53, WTp53)联合顺铂等化疗可取得较好的抗癌效果^[28, 29]^。增加细胞内的p53浓度能够扩大顺铂和紫杉醇在NSCLC中的化疗效果。但也有研究表明单独转染WTp53不足以很大程度上改变肺癌细胞系的化疗敏感性，而且，WTp53可诱导环氧化酶的生成，后者表现出抑制化疗诱导的凋亡^[30, 31]^。W Tp53第4外显子72密码子处存在单个核苷酸多态性(single-nucleotide polymorphism, SNP)，对肺癌的化疗敏感性有一定影响，临床研究发现，携带p53 72PRO等位基因肺癌患者的化疗敏感性明显高于携带ARG/ARG基因型的患者^[32]^。

### 2.2 P63、p73与肺癌的化疗敏感性

TAp63α在化疗敏感性上占据一定的地位，TAp63α可以被多种药物诱导凋亡，研究显示，TAp63α的过表达与肿瘤的化疗敏感性有关，TAp63α的下调会导致化疗抵抗。ΔNp63可直接影响一些头颈部肿瘤对顺铂的化疗敏感性，并可以作为重要的预后因子。有关p63与肺癌化疗敏感性的研究目前开展得较少^[24]^。

P73被证实了可以被多种细胞毒性药物诱导使细胞发生凋亡，如喜树碱、依托泊苷、顺铂、阿霉素及紫杉醇等。TAp73可被MTp53结合而发生显性失活，也能被siRNA抑制而发生失活，导致人类肿瘤细胞的化疗抵抗和造成细胞的转化。运用siRNA减少MTp53能增强肿瘤的化疗敏感性，支持了MTp53能通过中和TAp73来诱导化疗抵抗^[33]^。研究显示70%的人类肿瘤可表现出ΔNp73的上调，ΔNp73通过络合物的形成来抑制WTp53及TAp73的诱导凋亡功能，而通过特异性siRNA来抑制ΔNp73的表达能够提高化疗的敏感性。P73β的过表达能通过诱导p53依赖的凋亡通路来提高化疗敏感性，所以可以用于治疗p53抵抗的肿瘤^[34]^。

## 3 P53家族成员与肺癌的靶向治疗

放疗和化疗目前还是肺癌的主要治疗手段，但效果欠佳，肺癌靶向治疗是一个很有希望的研究方向，各种恢复失活p53的转录激活功能的研究目前正在广泛开展。一些策略正研究作用于失活/被抑制的p53，使其恢复WTp53的功能，如使突变的p53重新获得转录激活的功能。这些药物包括一些短多肽、小分子药物及重组腺病毒。

### 3.1 解除MDM2对p53、p73的抑制作用

E3泛素化连接酶MDM2是p53的负性调节蛋白质，在一些肉瘤中经常过表达。MDM2可抵消p53的肿瘤抑制功能，主要通过三个方面的机制：①与p53直接结合，阻碍后者的转录激活功能；②通过核输出p53使p53局限化；③介导p53的泛素化降解^[35, 36]^。一系列MDM2的小分子抑制剂(molecule inhibitor, MI)如Nutlins、Cis-imidazoline等，正研究用于结合到MDM2上而干扰后者对p53的抑制作用，使肿瘤细胞的被抑制的WTp53可以重新激活^[37]^。Nutlin-3可在紫杉醇的化疗中保护正常细胞而选择性地增加肺癌细胞的凋亡。Nutlin-3能下调肺癌细胞中TNF-α对NFκB的诱导激活，增强p53的转录激活功能而降低癌细胞的生存能力^[38]^。Nutlin还能增加肺癌的放疗敏感性^[39]^。MI-43在低浓度中可诱导p21而造成细胞在G_1_期或G_2_期阻滞，在高浓度时可以通过激活*puma*/*noxa*而诱导凋亡，呈剂量依赖性^[40]^。MDM2同样可影响p63、p73的功能，MDM2不介导对p73的降解，但可抑制p73的转录激活功能，基于Nutlin-3在MTp53的肿瘤细胞中仍然能抑制细胞生长和诱导凋亡^[41]^，所以可以假设Nutlin-3同样可以把p73从后者与MDM2的结合中释放出来，而激活下游的*noxa*、*puma*和*p21*，增强凋亡和细胞阻滞。同时，Nutlin-3可以提高缺乏p53的神经母细胞瘤对阿霉素的化疗敏感性^[42]^。

### 3.2 短多肽/小分子药物对突变*p53*的重激活作用

一些短多肽可以令一些未折叠的MTp53实现重折叠而使后者的活性与WTp53的活性一致。CDB3可重折叠MTp53但不影响p53对DNA的结合，这种重折叠可以是直接结合，也可以是通过分子伴侣实现^[43]^。在肺癌细胞系中，p53结合蛋白质2/p53相关凋亡刺激蛋白质(p53 binding protein 2/apoptosis stimulating protein of p53, 53BP2/ASPP)可以增强p53的转录激活的功能而与p53的状态无关，同时可以增强癌细胞对紫外线和X-线的敏感性^[44]^。53BP2L/ASPP2可特异性地增强p53-诱导的凋亡通过提高p53启动子和促凋亡基因的DNA结合及转录激活功能，如*bax*^[45]^。在肺癌细胞中某些热点突变的p53可以抑制p73介导的转录激活功能^[46]^，在其它肿瘤细胞研究中，一些短多肽可以把与MTp53结合的p73游离出来，这一策略可以提高p73的促凋亡活性，并且提高化疗敏感性^[33, 43]^，可设想研究用于肺癌细胞治疗上。小分子药物如Ellipticine、p53-依赖性重激活和诱导大量凋亡(p53-dependent reactivation and induction of massive apoptosis, PRIMA-1)、p53重激活和诱导细胞凋亡(reactivation of p53 and induction of tumour cell apoptosis, RITA)等可恢复MTp53的转录活性。PRIMA-1可抑制表达MTp53的癌细胞生长，激活p53靶向的*mdm2*、*p21*、*puma*等基因^[43]^。在肺癌细胞系中，PRIMA-1可增强p53依赖的细胞凋亡或协同化疗药物的效果^[47, 48]^。

### 3.3 重组腺病毒的靶向治疗

针对*p53*突变或者缺失的癌细胞，可用腺病毒为载体将WTp53输入，使癌细胞重新获得p53介导的抗癌能力。肺癌细胞中的*p53*突变广泛存在，目前，重组人p53腺病毒结合化疗治疗肺癌已经进行了临床前和临床测试^[49]^，细胞系研究显示，重组人p53腺病毒注射液能抑制肺腺癌细胞的生长，并不受内源性p53状态的影响。它与抗癌药顺铂联用能明显增加肺腺癌细胞的化疗敏感性^[50]^。一项涉及49例NSCLC的临床研究^[51]^显示，19例患者运用支气管动脉融合结合重组腺病毒治疗比49例单纯用支气管动脉融合治疗能够提高患者的生活质量和延迟疾病的进展。

综上所述，p53家族成员之间的的作用与联系为肺癌的发生，化疗敏感性及靶向治疗上提供了新的研究方向，在细胞系和肺癌标本上做基因或蛋白质水平的分子基础、调控作用的研究已经广泛开展，p53的基因治疗已应用到肺癌的临床治疗中，但是，还有很多问题需要深入解决。例如，p53家族里各个亚型之间的相互作用，基因多态性的影响如何等。因此，对于p53家族成员与肺癌的关系的研究有待进一步深化和广泛化。
